# Prognostic Value of Late Enhanced Cardiac Magnetic Resonance Imaging Derived Texture Features in Dilated Cardiomyopathy Patients With Severely Reduced Ejection Fractions

**DOI:** 10.3389/fcvm.2021.766423

**Published:** 2021-12-17

**Authors:** Shenglei Shu, Cheng Wang, Ziming Hong, Xiaoyue Zhou, Tianjng Zhang, Qinmu Peng, Jing Wang, Chuansheng Zheng

**Affiliations:** ^1^Department of Radiology, Union Hospital, Tongji Medical College, Huazhong University of Science and Technology, Wuhan, China; ^2^Hubei Province Key Laboratory of Molecular Imaging, Wuhan, China; ^3^Department of Cardiology, Institute of Cardiology, Union Hospital, Tongji Medical College, Huazhong University of Science and Technology, Wuhan, China; ^4^School of Electronic Information and Communications, Huazhong University of Science and Technology, Wuhan, China; ^5^MR Collaboration, Siemens Healthineers, Shanghai, China; ^6^Philips Healthcare, Guangzhou, China

**Keywords:** cardiac magnetic resonance, texture features, dilated cardiomyopathy, prognosis, survival

## Abstract

**Background:** Late enhanced cardiac magnetic resonance (CMR) images of the left ventricular myocardium contain an enormous amount of information that could provide prognostic value beyond that of late gadolinium enhancements (LGEs). With computational postprocessing and analysis, the heterogeneities and variations of myocardial signal intensities can be interpreted and measured as texture features. This study aimed to evaluate the value of texture features extracted from late enhanced CMR images of the myocardium to predict adverse outcomes in patients with dilated cardiomyopathy (DCM) and severe systolic dysfunction.

**Methods:** This single-center study retrospectively enrolled patients with DCM with severely reduced left ventricular ejection fractions (LVEFs < 35%). Texture features were extracted from enhanced late scanning images, and the presence and extent of LGEs were also measured. Patients were followed-up for clinical endpoints composed of all-cause deaths and cardiac transplantation. Cox proportional hazard regression and Kaplan–Meier analyses were used to evaluate the prognostic value of texture features and conventional CMR parameters with event-free survival.

**Results:** A total of 114 patients (37 women, median age 47.5 years old) with severely impaired systolic function (median LVEF, 14.0%) were followed-up for a median of 504.5 days. Twenty-nine patients experienced endpoint events, 12 died, and 17 underwent cardiac transplantations. Three texture features from a gray-level co-occurrence matrix (GLCM) (GLCM_contrast, GLCM_difference average, and GLCM_difference entropy) showed good prognostic value for adverse events when analyzed using univariable Cox hazard ratio regression (*p* = 0.007, *p* = 0.011, and *p* = 0.007, retrospectively). When each of the three features was analyzed using a multivariable Cox regression model that included the clinical parameter (systolic blood pressure) and LGE extent, they were found to be independently associated with adverse outcomes.

**Conclusion:** Texture features related LGE heterogeneities and variations (GLCM_contrast, GLCM_difference average, and GLCM_difference entropy) are novel markers for risk stratification toward adverse events in DCM patients with severe systolic dysfunction.

## Introduction

Idiopathic dilated cardiomyopathy (DCM) is one of the most common non-ischemic cardiomyopathies, which is associated with a poor prognosis due to sudden cardiac death and heart failure (HF) (1, 2). Left ventricular systolic function (left ventricular ejection fraction, LVEF) has been the dominant factor for risk stratification and survival prognosis in DCM patients with HF (3–5). However, risk stratification in DCM patients with severely reduced LVEF remains challenging. Myocardial fibrosis evaluated by late gadolinium enhancement (LGE) cardiac magnetic resonance (CMR) imaging showed significant value for risk stratification in patients with DCM (6). Both the presence and the extent of LGE are significantly associated with adverse outcomes in patients with DCM (7, 8). However, recent studies have demonstrated that different LGE distribution patterns within the myocardium could also affect prognosis in patients with DCM (9–11).

Texture analysis is a quantitative imaging-processing method, which can detect subtle pixel formation features that cannot be observed by direct visual inspection (12, 13). Following the application of texture analysis in oncology studies (14, 15), texture features were also found to provide valuable information in the diagnosis and prognosis of ischemic and non-ischemic cardiomyopathies (16–18). We hypothesize that texture features extracted from late enhanced images may provide incremental information regarding ordinary LGE analysis for prognosis among patients with DCM. We focused on DCM patients with severely impaired systolic function (LVEF < 35%), who had a high prevalence of LGE presence in the CMR study with an increased risk of adverse cardiac events. We aimed to evaluate texture characteristics extracted from LGE–CMR images in DCM patients with severely reduced LVEF to assess its prognostic value for further stratifying this subgroup of patients.

## Materials and Methods

### Study Population

A retrospective study was conducted at a local institute on patients enrolled between June 2014 and April 2020 through searching the Picture Archiving and Communication Systems and medical records. Our study was conformed to the principles outlined in the Declaration of Helsinki and was approved by the local ethics committee. Due to the retrospective nature of the study, written informed consent from patients was waived by the local ethical committee.

Patients referred to contrast-enhanced CMR examination for evaluation of cardiomyopathic etiologies at the local institute were retrospectively reviewed. By reviewing medical records, those who had been previously diagnosed with DCM and with LVEF < 35% were finally included. The diagnosis of DCM was made according to the criteria of the WHO/International Society and Federation of Cardiology (19). Exclusion criteria were as follows: (1) significant ischemic cardiomyopathy, defined as >50% stenosis of major coronary arteries, previous coronary revascularization, or myocardial infarction; (2) moderate-to-severe valvulopathy; (3) hypertrophic cardiomyopathy, amyloid cardiomyopathy, and other infiltrative heart diseases; (4) evident artifacts on CMR images due to either arrhythmia, difficulties with breath-hold, or implanted devices; and (5) patients who cannot be followed for survival outcome were also excluded. In keeping with the guidelines, patients showing LGE infarct patterns on late enhancement images were also excluded.

### CMR Examination

Cardiac magnetic resonance was performed at 1.5 or 3.0 T MR scanners (MAGNETOM Aera or MAGNETOM Skyra, Siemens, Erlangen, Germany) with 12-channel surface-phased array coils. The standard acquisition protocol included steady-state free-precession cine-sequences in 3 long-axis views and continuous short-axis views, covering the entire LV and late enhancement sequences after administrating the gadolinium contrast agent. Typical imaging parameters for cine imaging were as follows: field of view 415 × 340 mm^2^, matrix 256 × 256, slice thickness 8 mm, slice gap 1.6 mm, temporal resolution < 45 ms, number of calculated cardiac phases 25. LGE images were acquired 10–15 min (median time interval 12.4 min) after cumulative intravenous administration of 0.2 mmol/kg (median amount 13.0 mmol) of gadopentetate dimeglumine (Magnevist, Bayer, Germany) or gadobutrol (Gadovist, Bayer, Germany), with the same positions used for cine-CMR imaging. After selecting an inversion time for optimal normal myocardium nulling with an inversion time scout sequence, a phase-sensitive inversion recovery technique was used to acquire LGE images with the following parameters: field of view, 400 × 360 mm^2^; matrix, 256 × 256; slice thickness, 8 mm; slice gap, 1.6 mm.

### Conventional CMR Image Analysis

The conventional analysis was performed offline using dedicated commercially available software (CMR42, Circle Cardiovascular Imaging Inc., Calgary, Canada) following standardized recommendations by researchers blinded to clinical data. Conventional parameters derived from cine images included EF of bilateral ventricles, end-systolic and end-diastolic volumes of bilateral ventricles indexed to body surface area, and LV mass index. The presence and extent, and LGE distributions were evaluated by two senior operators with a consensus. An LGE was defined as an area with a signal intensity >6 standard deviations above the mean normal myocardial signal intensity derived in a remote location using the same short-axis slice. After drawing the contours of the epi- and endomyocardium, the extent of LGE was calculated as the percentage volume of enhanced myocardium accounting for the whole left ventricular myocardium. The LGE distribution was classified into different patterns, including septal, free-wall, or as involving both septal and free-wall.

### LGE Images Texture Analysis

**Figure 1:**
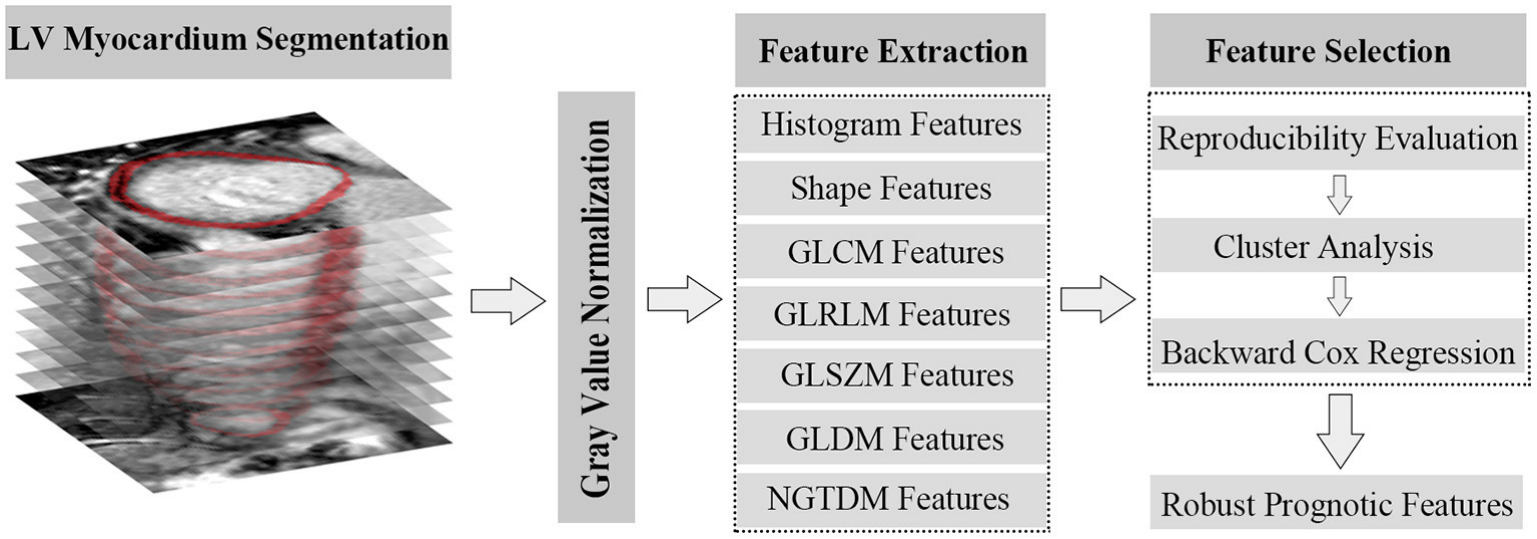
Flowchart of textures extraction and selection.

In late enhanced scanning, the LV myocardium was segmented and extracted in continuous short-axis views according to the contours drawn in the conventional analysis process. The process of texture feature extraction was achieved with a radiomic python toolbox named pyradiomics (20). The original images were first normalized by ranging gray pixel values from 0 to 255 and then discretized them (with bandwidth = 5) into 52 gray levels. Texture features were extracted from the processed images, including features from the categories of histogram, shape, gray-level co-occurrence matrix (GLCM), gray-level run-length matrix (GLRLM), gray-level size-zone matrix (GLSZM), gray-level dependence matrix (GLDM), and neighboring gray-tone difference matrix (NGTDM). To assess the intra-/inter-reader agreement of the feature analyses, the segmentation of myocardial contour was performed again in all cases by the same researcher and the other researcher 2 weeks later. After assessing the reproducibility of extracted features, they were grouped by correlation. Further selection processes were implemented among each group to generate robust prognostic texture features (Figure 1).

### Follow-Up and Definition of Endpoints

All patients were followed-up *via* telephone in October 2020 by researchers blinded to the clinical information. The endpoint was defined as the composite of all-cause death and heart transplantation. The duration of follow-up was calculated from the baseline CMR study date to the occurrence of endpoint event or last contact with the patient. Baseline clinical information for each patient was retrieved from medical records.

### Statistical Analysis

Continuous variables were expressed as medians and interquartile ranges (IQRs). Categorical data were described as frequencies and percentages. For assessment-extracted texture features robustness, inter-/intraclass correlation (ICC) was analyzed based on different myocardial segmentation groups. The Spearman correlation coefficient with cluster analysis was performed to divide the texture features into different groups. Then, multivariate Cox proportional hazard regression analysis with backward stepwise selection was performed in each group to identify the non-redundancy representative features. This process was implemented again on all identified features to determine a non-redundant set of prognostic indicators.

Student's *t*-test, Wilcoxon–Mann–Whitney test, χ^2^ test, or Fisher's exact test were used to compare the differences between patients with and without endpoint events. Kaplan–Meier (K–M) curves and log-rank tests were used to analyze the association between baseline variables and cardiac events. For continuous variables, median values were chosen for transforming into categorical data. Cox proportional hazards regression models were used to estimate hazard ratios (HR) and 95% confidence intervals (95% CIs) were used to assess the association between baseline variables and endpoint events in both univariate and multivariable analyses. The prognostic abilities of constituted models were assessed by calculating the concordance probability (C index). A two-sided value of *p* < 0.05 was considered statistically significant. All analyses were performed using SPSS software (version 22.0, IBM, Armonk, NY, USA).

## Results

### Clinical Demographics and Survival Outcomes

**Figure 2:**
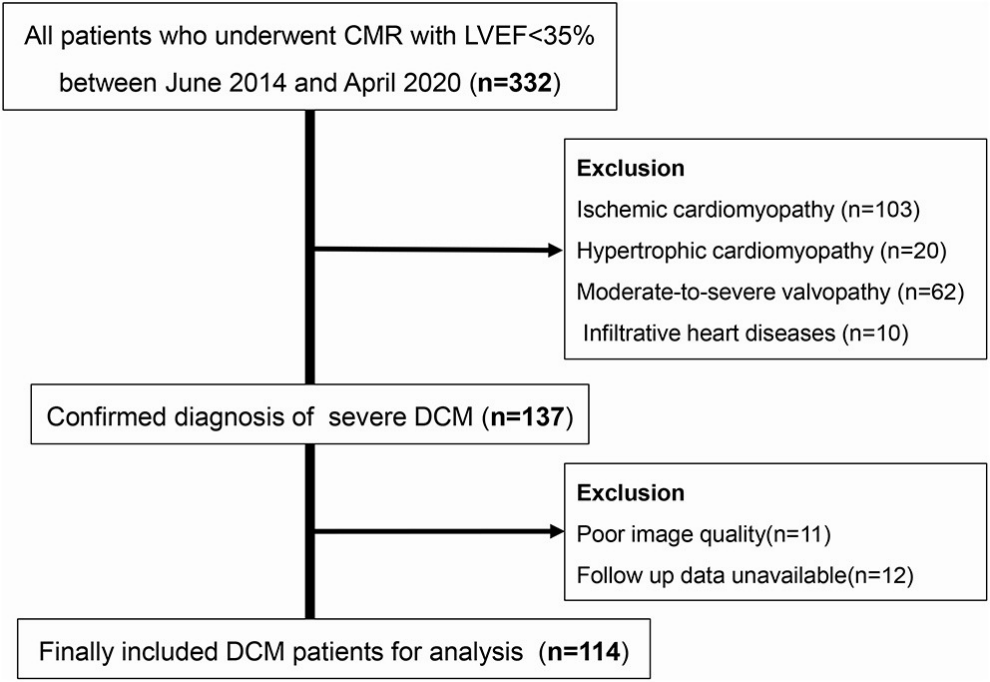
Flowchart of patients selection.

A total of 114 patients were included after multiple selection processes (Figure 2). The median age was 47.5 years and the total number of women was 37. During a median follow-up of 504.5 days, 29 endpoint events were documented, including 12 deaths and 17 cardiac transplantations. Baseline clinical characteristics were compared between patients with endpoint events and those who survived (Table 1). There were no significant differences between groups with regard to age, sex, family history, and underlying diseases. In addition, patients having adverse events had lower blood pressure and worse heart function grades. The duration of the QRS wave on the electrocardiogram was also longer in the group with endpoint events. Except for beta-blockers, there was no difference in drug medications between the groups.

**Table 1:** Baseline clinical characteristics.

	**All patients** **(*n* = 114)**	**Patients with endpoints (*n* = 29)**	**Patients without endpoints** **(*n* = 85)**	***P*-value**
**Clinical demographics**				
Age, years	47.5 (35.8, 57.0)	56.0 (36.0, 59.0)	47.0 (33.5, 56.0)	0.280
Female, *n* (%)	37 (32.5%)	7 (24.1%)	30 (35.3%)	0.268
BMI (kg/m^2^)	24.1 (21.0, 26.4)	22.5 (19.3, 25.3)	24.6 (21.4, 26.7)	0.027
Family history	13 (11.4%)	2 (6.9%)	11 (12.9%)	0.51
Hypertension, *n* (%)	23 (20.2%)	4 (13.8%)	19 (22.4%)	0.426
Diabetes mellitus, *n* (%)	16 (14.0%)	6 (20.7%)	10 (11.8%)	0.232
Tobacco, *n* (%)	39 (34.2%)	9 (31.0%)	30 (35.3%)	0.821
Alcohol, *n* (%)	20 (17.5%)	5 (17.2%)	15 (17.6%)	>0.99
Dyslipidemia, *n* (%)	17 (14.9%)	5 (17.2%)	12 (14.1%)	0.764
SBP (mmHg)	115 (102, 130)	100 (94, 114)	121 (109, 135)	<0.001
DBP (mmHg)	72 (64, 88)	65 (60, 74)	77 (67, 88)	0.005
**NYHA functional class**				
I, *n* (%)	1 (0.9%)	0 (0%)	1 (1.3%)	0.007
II, *n* (%)	29 (27.1%)	7 (24.1%)	22 (28.2%)	
III, *n* (%)	47 (43.9%)	7 (24.1%)	40 (51.3%)	
IV, *n* (%)	30 (28.0%)	15 (51.7%)	15 (19.2%)	
**Electrocardiogram**				
Heart rate (bpm)	89 (72, 100)	76 (67, 97)	89 (74, 101)	0.210
AVB, *n* (%)	3 (2.7%)	1 (3.6%)	2 (2.4%)	>0.99
LBBB, *n* (%)	15 (13.6%)	4 (14.3%)	11 (13.4%)	>0.99
RBBB, *n* (%)	4 (3.6%)	1 (3.6%)	3 (3.7%)	>0.99
QRS duration (ms)	100.0 (91.5, 116.5)	106.0 (100.0, 132.0)	100.0 (90.0, 110.0)	0.021
QTc interval (ms)	438.5 (413.75, 466.5)	450 (420, 485)	436 (412.25, 464.5)	0.163
**Cardiac medication**				
Beta-blockers, *n* (%)	87 (76.3%)	18 (62.1%)	69 (81.2%)	0.037
ACEI/ARB, *n* (%)	35 (30.7%)	11 (37.9%)	24 (28.2%)	0.328
Diuretics, *n* (%)	43 (37.7%)	15 (51.7%)	28 (32.9%)	0.072
Spironolactone, *n* (%)	74 (64.9%)	21 (72.4%)	53 (62.4%)	0.327
Digoxin, *n* (%)	37 (32.5%)	10 (34.5%)	27 (31.8%)	0.787

*BMI, body mass index; SBP, systolic blood pressure; DBP, diastolic blood pressure; NYHA, new york heart association; AVB, atrioventricular block; LBBB, left branch bundle block; RBBB, right branch bundle block; ACEI, angiotensin-converting-enzyme inhibitor; ARB, angiotensin II receptor blockers*.

### CMR Characteristics

Baseline CMR parameters of both the groups are shown and compared in Table 2. The median LVEF in all patients was severely impaired (14.0%) without a significant difference between the event and event-free groups (13.7% vs. 14.9%, *p* = 0.873). However, the RVEF value was higher in patients with adverse outcomes. Indexed parameters of ventricular volume and LV mass showed no differences between the groups. The presence of LGEs was prevalent in the study cohort (83.3%), and there was no significant difference between the events group and those without cardiac events. In addition, the locations of LGE on LV myocardium did not show a significant difference between the groups. However, the extent of LGE areas, accounting for the entire LV myocardium, was much more significant in patients who experienced adverse events (3.65% vs. 1.61%, *p* = 0.001).

**Table 2:** Baseline cardiac magnetic resonance (CMR) parameters.

**CMR parameters**	**All patients** **(*n* = 114)**	**Patients with endpoints (*n* = 29)**	**Patients without endpoint** **(*n* = 85)**	***P*-value**
LV EF (%)	14.0 (10.7, 19.2)	13.7 (11.0, 19.2)	14.9 (10.3, 19.5)	0.873
LV EDVI (ml/m^2^)	175.8 (147.7, 214.2)	161.6 (133.8, 203.1)	185.4 (150.8, 219.0)	0.116
LV ESVI (ml/m^2^)	149.4 (120.5, 191.5)	148.4 (110.8, 176.0)	154.2 (124.5, 195.8)	0.218
LV mass index (g/m^2^)	88.6 (74.6, 105.8)	89.5 (77.0, 95.8)	88.1 (73.7, 109.0)	0.977
RV EF (%)	18.8 (9.3, 33.3)	29.8 (14.2, 38.1)	16.0 (8.5, 29.0)	0.034
RV EDVI (ml/m^2^)	88.7 (65.3, 114.4)	89.7 (64.3, 115.8)	88.7 (65.6, 113.0)	0.925
RV ESVI (ml/m^2^)	66.0 (46.4, 94.6)	65.7 (43.7, 92.4)	66.2 (51.6, 96.6)	0.382
Presence of LGE, *n* (%)	95 (83.3%)	27 (93.1%)	68 (80.0%)	0.149
LGE location				0.203
Absent	19 (16.7%)	2 (6.9%)	17 (20%)	
Only septal LGE	10 (8.8%)	2 (6.9%)	8 (9.4%)	
Only free-wall LGE	4 (3.5%)	0 (0.0%)	4 (4.7%)	
Septal & free-wall LGE	81 (71.1%)	25 (86.2%)	56 (65.9%)	
LGE extent	2.21 (0.68, 4.86)	3.65 (1.29, 11.13)	1.61 (0.51, 3.09)	0.001

*CMR, cardiac magnetic resonance; LV, left ventricle; EF, ejection fraction; EDVI, indexed end-diastolic volume; ESVI, indexed end-systolic volume; RV, right ventricle; LGE, late gadolinium enhancement*.

### Texture Analysis

A total of 58 texture features with good reproducibility, defined as ICCs higher than 0.7, w included in the final analysis. There were six first-order statistical features, 18 GLCM features, 11 GLRLM features, 10 GLSZM features, 10 GLDM features, and 3 NGTDM features. They were divided into 10 different groups by cluster analysis; all pairs of features within each group had Spearman correlation coefficients >0.4. According to the prognostic value of the texture features of adverse outcome, the backward stepwise Cox method was used to select robust texture features among each group. A total of 20 texture features was chosen from 10 different groups. To further minimize the number of meaningful features and find out more robust markers, backward Cox analysis was implemented again between these candidate features, and three features (GLCM_contrast, GLCM_ difference average and GLCM_difference entropy) were finally selected. Their basic characteristics are described in Table 3.

**Table 3:** Characteristics of employed texture features for survival analysis.

	**Intuitive description**	**Median (IQR)**			***P*-value**
		**All patients**	**Events group**	**Event-free group**	
GLCM_contrast	It is a measure of the local intensity variation, favoring values away from the diagonal.	20.923 (16.191, 25.836)	24.719 (19.213, 32.556)	19.881 (15.969, 24.807)	0.007
GLCM_difference average	It measures the relationship between occurrences of pairs with similar intensity values and occurrences of pairs with differing intensity values.	3.275 (2.825, 3.688)	3.502 (3.187, 4.065)	3.216 (2.786, 3.646)	0.011
GLCM_difference entropy	It is a measure of the randomness/variability in neighborhood intensity value differences.	3.260 (3.081, 3.413)	3.361 (3.223, 3.562)	3.235 (3.041, 3.402)	0.007

*IQR, Interquartile range; GLCM, gray-level co-occurrence matrix*.

### Survival Analysis

**Figure 3:**
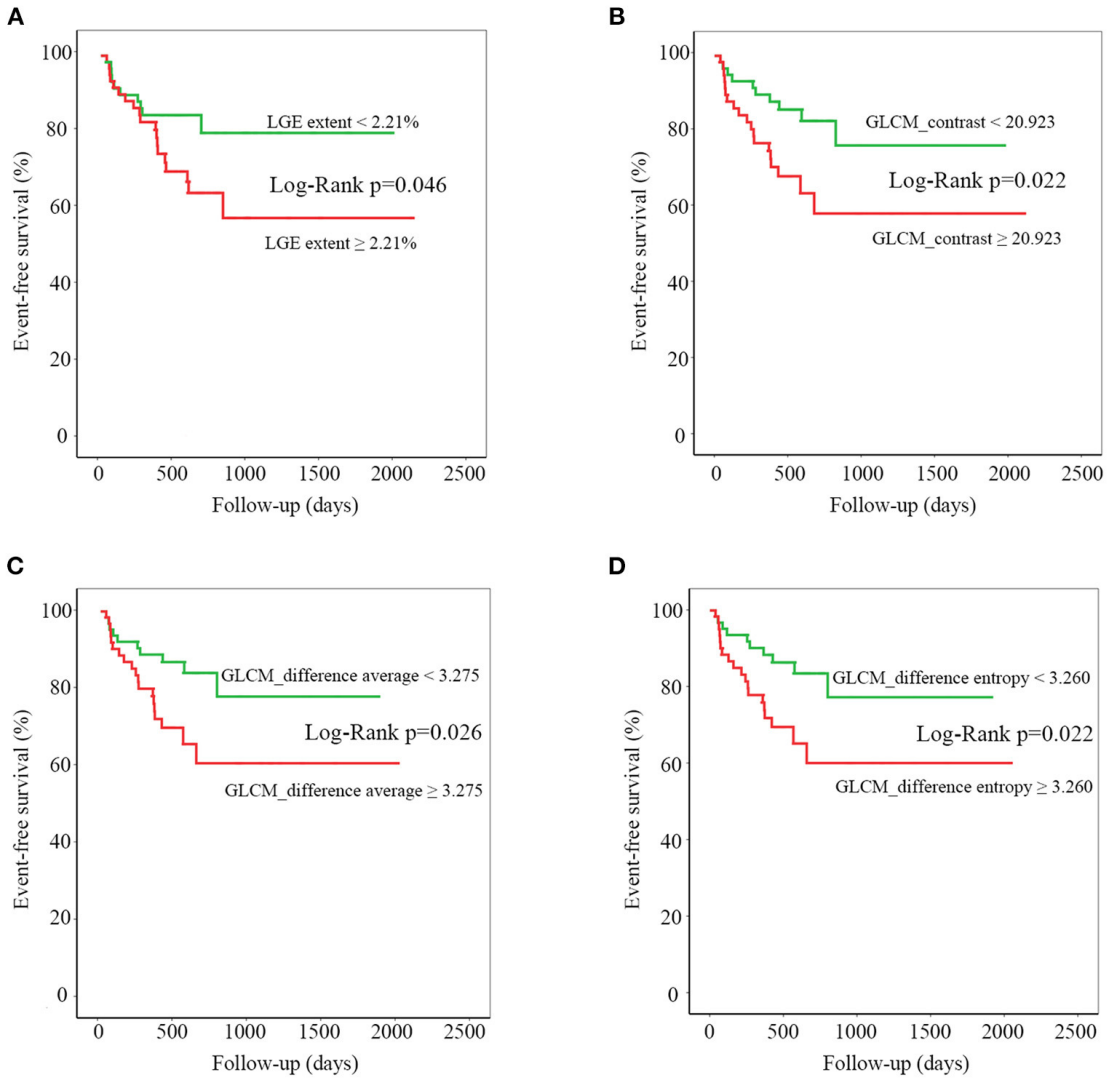
Kaplan-Meier curves for late gadolinium enhancement (LGE) extent **(A)**, gray-level cooccurrence matrix (GLCM)_contrast **(B)**, GLCM_difference average **(C)**, and GLCM_difference entropy **(D)**. For each texture feature, median value was chosen to divide the entire patient population into two parts. Statistical difference between curves was measured using the log-rank test.

The baseline clinical variables, conventional CMR parameters, and texture features derived from late enhanced images were analyzed to evaluate the association with adverse outcomes (Table 4). Among the clinical variables, BMI, blood pressure, heart functional classification, diuretics, and beta-blockers medications were associated with endpoint events. Regarding conventional CMR parameters, RVEF, and LGE extent, especially the latter one, was shown to be significantly associated with adverse outcome. However, the presence and location of LGE showed no association with adverse events. All three included texture features have predicted the adverse outcome in our study cohort (Table 4). Dividing by the median values, LGE extent, GLCM_contrast, GLCM_difference average, and GLCM_difference entropy showed significant associations with adverse events on K–M survival curves (log-rank *p* < 0.05 for all, Figure 3).

**Table 4:** Univariable Cox regression analysis of baseline clinical variables.

	**HR**	**95% CI**	***P*-value**
**Clinical demographics**			
Age, years	0.940	0.973–1.030	0.940
Gender (Female)	1.528	0.652–3.582	0.329
BMI (kg/m^2^)	0.892	0.836–0.925	0.001
Hypertension	0.600	0.208–1.725	0.343
Diabetes mellitus	1.533	0.624–3.767	0.352
Tobacco	0.906	0.412–1.991	0.806
Alcohol	0.953	0.363–2.501	0.922
Dyslipidemia	1.263	0.482–3.312	0.635
SBP (mmHg)	0.953	0.930–0.976	0.000
DBP (mmHg)	0.963	0.936–0.990	0.008
NYHA function class	2.03	1.36–3.04	0.000
**ECG**			
Heart rate (bpm)	0.988	0.970–1.008	0.233
AVB	1.531	0.208–11.296	0.676
LBBB	1.123	0.389–3.241	0.830
RBBB	1.235	0.167–9.150	0.836
QRS duration (ms)	1.011	0.998–1.024	0.096
QTc interval (ms)	1.003	0.997–1.009	0.366
**Cardiac medication**			
Beta-blockers	0.393	0.183–0.841	0.016
ACEI/ARB	1.301	0.610–2.779	0.496
Diuretics	2.154	1.038–4.469	0.035
Spironolactone	1.590	0.703–3.594	0.265
Digoxin	1.064	0.494–2.291	0.875
**Cardiac MRI**			
LV EF (%)	0.999	0.945–1.056	0.963
LV EDVI (ml/m^2^)	0.996	0.990–1.002	0.224
LV ESVI (ml/m^2^)	0.996	0.990–1.003	0.257
LV mass index (g/m^2^)	0.999	0.986–1.012	0.849
RV EF (%)	1.025	1.000–1.050	0.046
RV EDVI (ml/m^2^)	0.998	0.987–1.009	0.752
RV ESVI (ml/m^2^)	0.994	0.983–1.005	0.283
LGE presence	3.247	0.771–13.680	0.109
LGE location	1.513	0.991–2.310	0.055
LGE extent	1.070	1.039–1.102	0.000
**Texture features**			
GLCM_contrast	1.072	1.031–1.114	0.000
GLCM_difference average	2.482	1.424–4.327	0.001
GLCM_difference entropy	12.748	2.692–60.371	0.001

*HR, hazard ratio; CI, confidence interval and the rest abbreviations are same as those in Tables 1, 2*.

Considering that a limited number of patients experienced endpoint events, and after assessing collinearity and evaluating clinical interpretability, systolic blood pressure (SBP) and LGE extents were included in multivariable Cox regression analysis with selected texture features. When incorporated into the multivariable Cox regression model, each texture feature was identified as a significant independent predictor of adverse events (*p* < 0.05 for all three features, Table 5). All three multivariable Cox regression models containing SBP, LGE extent, and one of the selected texture features showed favorable prognostic value indicated by high C-indices (0.778, 0.774, and 0.775 for three models).

**Table 5:** Multivariable Cox regression analysis incorporating texture features.

	**GLCM_contrast**		**GLCM_difference average**		**GLCM_difference entropy**	
	**HR (95% CI)**	***P*-value**	**HR (95% CI)**	***P*-value**	**HR (95% CI)**	***P*-value**
SBP	0.960 (0.936–0.985)	0.002	0.961 (0.936–0.986)	0.002	0.961 (0.937–0.986)	0.002
LGE extent	1.051 (1.016–1.086)	0.004	1.050 (1.015–1.085)	0.004	1.049 (1.015–1.085)	0.005
GLCM_contrast	1.061 (1.014–1.110)	0.010	–	–	–	–
GLCM_difference average	–	–	2.151 (1.119–4.135)	0.022	–	–
GLCM_difference entropy	–	–	–	–	7.645 (1.287–45.417)	0.025

*HR, hazard ratio; CI, confidence interval*.

## Discussion

Novel features derived from CMR images can provide prognostic value for adverse events in patients with severe DCM. These features are of significant use in improving the outcomes among high-risk patients. In this preliminary study, we found that some texture features related to myocardial heterogeneities and variations were strongly associated with adverse events in DCM patients with severely reduced LVEF. Along with the LGE extent and other clinical variables, these novel markers offered a new method to evaluate adverse events in patients with DCM.

Fibrosis replacement of normal myocardial tissue is an important cause of progressive ventricular dysfunction and arrhythmic events in non-ischemic cardiomyopathic patients (21, 22). In histological studies, LGE detected on CMR imaging has been validated to correspond to myocardial fibrosis in patients with DCM (23). Previous studies demonstrated that the presence and the extent of LGE showed a significant value in predicting adverse events (7, 24). Recently, Halliday et al. found that, in addition to presence and extent, the location and pattern of LGE distribution also provided prognostic value for endpoint events (9). Similar to previous studies, our study reported that the extent of LGE was much more significant in patients with adverse outcomes. It showed a significant value in predicting the adverse events in DCM patients with severely reduced LVEF. However, visual classification of LGE distribution based on their location had minimal value in differentiation and prognostication in our study. The location pattern of LGE in LV myocardium neither showed any difference between groups nor provided a prognostic value for adverse events.

Microscopically, the myocardial interstitial fibrosis pattern is highly heterogeneous in non-ischemic cardiomyopathic patients. More than three types of myocardial fibrosis: diffuse microscars, perivascular fibrosis, perimysial, and endomysial fibrosis could exist in various patients or the same patient at different stages of the disease (25, 26). The microscopic differences between multiple myocardial fibrosis patterns may contribute to different myocardial structural abnormalities with varying extent of risk for adverse events (27). Thus, novel markers that assess heterogeneities of myocardial fibrosis detected as LGEs on CMR imaging, will be of significant value for risk stratification in patients with DCM (28). In a retrospective study, Muthalaly et al. found that a first-order texture feature, LV entropy, showed significant value for risk stratification toward ventricular arrhythmia in patients with DCM (29). In another study of hypertrophic cardiomyopathy patients, Cheng et al. demonstrated that features extracted from the histogram, GLCM, and GLRLM matrixes could offer incremental value to LGE extent in predicting the adverse events (30). In our study, the three selected texture features of late enhanced images, contrast, difference average, and difference entropy of the LV myocardium GLCM matrix, were positively related to heterogeneities and variations of myocardial fibrosis. The increase of myocardial fibrosis, particularly the scattered distributed pattern, increases the contrast between neighboring voxels and disrupts the uniformity of regional intensity, thus leading to a higher difference average and difference entropy values. As shown in our study, these parameter values were significantly higher in patients with adverse outcomes. Even after combining with the LGE extent and other clinical risk factors, they remained independent risk factors for adverse outcomes and provided incremental value for risk stratification.

Specifically, we focused on DCM patients with severely reduced LVEF, in whom LGE is highly prevalent. The extent of LGE tends to increase dramatically in the end-stage among patients with DCM, and they are associated with higher rates of adverse outcomes (31). According to the study of Halliday et al., the prognostic value of LGE extent is limited when there is a large amount of LGE (9). The texture features derived from late enhanced images may act as potential markers for risk stratification and outcome prognostication in such situations. The results of this preliminary study demonstrated that texture features of late enhanced images could be promising predictors for prognostic assessment.

There are several limitations to our study. First, as it was a single-center study, the number of included patients was limited and the follow-up period was short. Additionally, the research was conducted at a tertiary center with easily accessible transplantation treatment, which might have increased the event rate. However, the endpoint-event rates were low; only 12 deaths and 17 cardiac transplantations were reported, limiting the creation of a more sophisticated risk stratification model. Besides, the method of following patients with telephone calls may introduce systemic bias since some patients were excluded as they cannot be contacted.

As for the imaging acquirement, images from scanners with different static field strengths (1.5 and 3.0 T) and contrast agents were analyzed together with a normalization process before feature extraction in our study. Additionally, the minimal differences during late enhanced image acquisitions that affect the texture features, such as delay time after contrast injection and inversion time selection are unknown. In our study, we compared the texture features between patients examined with different scanners and patients who received different contrast agents. We found that the three important texture features (GLCM_contrast, GLCM_difference average, and GLCM_difference entropy) that are closely related to survival outcome showed no differences between the patient groups. Specifically, there is no significant differences between patients when performed on 1.5T (*n* = 66) and 3.0 T (*n* = 48) scanners (GLCM_contrast: 3.152 vs. 3.382, *p* = 0.239; GLCM_difference average: 3.265 vs. 3.402, *p* = 0.288; GLCM_difference entropy: 3.227 vs. 3.290, *p* = 0.216) and between patients administrated with Magnevist (*n* = 67) and Gadovist (*n* = 47) (GLCM_contrast: 20.703 vs. 22.171, *p* = 0.338; GLCM_difference average:3.265 vs. 3.406, *p* = 0.273; GLCM_difference entropy:3.232 vs. 3.284, *p* = 0.312). The median time interval between contrast administration and image acquisition was 12.40 min, and there was no significant difference between the patients group (12.43 vs. 12.37, *p* = 0.838). The median amount of contrast agent used for whole cohort was 13 mmol and there was no significant difference between the patients group (13.4 vs. 12.8, *p* = 0.056). However, further studies are required to identify factors that influence the values of these texture features. Besides, though with fixed slice thickness and gap, the minimal variation in slice number between patients caused by the length of LV might also affect the texture values. Finally, as the final three texture features were selected through a statistical process, the true meaning of the selected texture features needs further investigation by correlating these features with histological results in patients with DCM.

## Conclusions

Texture features related to myocardial fibrosis heterogeneity (GLCM_contrast, GLCM_difference average, and GLCM_difference entropy) are novel markers associated with adverse outcome in DCM patients with severely reduced systolic function. They demonstrate a promising ability to predict adverse events beyond conventional LGE parameters in patients with severe DCM.

## Data Availability Statement

The raw data supporting the conclusions of this article will be made available by the authors, without undue reservation.

## Ethics Statement

The studies involving human participants were reviewed and approved by Ethics Committee of Tongji Medical College of Huazhong University of Science and Technology. Written informed consent from patients was waived by the ethical committee due to retrospective nature of the study.

## Author Contributions

CZ and JW contributed to the conception of the study. SS, CW, and ZH analyzed and interpreted the clinical data and imaging data and they were major contributors in writing the manuscript. QP helped perform the analysis with constructive discussions. XZ and TZ contributed greatly in writing and editing the manuscript. All authors read and approved the final manuscript.

## Funding

This study was funded by the National Natural Science Foundation of China (Grant No. 81701653).

## Conflict of Interest

XZ is employed by Siemens Healthineers and TZ was employed by Philips Healthcare. The remaining authors declare that the research was conducted in the absence of any commercial or financial relationships that could be construed as a potential conflict of interest.

## Publisher's Note

All claims expressed in this article are solely those of the authors and do not necessarily represent those of their affiliated organizations, or those of the publisher, the editors and the reviewers. Any product that may be evaluated in this article, or claim that may be made by its manufacturer, is not guaranteed or endorsed by the publisher.
